# Hermitian–Yang–Mills Connections on Collapsing Elliptically Fibered *K*3 Surfaces

**DOI:** 10.1007/s12220-021-00808-9

**Published:** 2022-01-07

**Authors:** Ved Datar, Adam Jacob

**Affiliations:** 1 Department of Mathematics, Indian Institute of Science, Bengaluru, India; 2 Department of Mathematics, UC Davis, One Shields Ave, Davis, CA USA

**Keywords:** Hermitian-Yang-Mills, Holomorphic degenerations, Elliptic fibrations, K3 surfaces, 53B35, 53C07, 53C26, 53C55

## Abstract

Let $X\rightarrow {{\mathbb {P}}}^1$ be an elliptically fibered *K*3 surface, admitting a sequence $\omega _{i}$ of Ricci-flat metrics collapsing the fibers. Let *V* be a holomorphic *SU*(*n*) bundle over *X*, stable with respect to $\omega _i$. Given the corresponding sequence $\Xi _i$ of Hermitian–Yang–Mills connections on *V*, we prove that, if *E* is a generic fiber, the restricted sequence $\Xi _i|_{E}$ converges to a flat connection $A_0$. Furthermore, if the restriction $V|_E$ is of the form $\oplus _{j=1}^n{\mathcal {O}}_E(q_j-0)$ for *n* distinct points $q_j\in E$, then these points uniquely determine $A_0$.

## Introduction

In this paper, we study degenerations of Hermitian–Yang–Mills connections on a *K*3 surface. We are motivated by the work of Gross–Wilson [1], and later Gross–Tosatti–Zhang [2, 3], who study Ricci-flat metrics on elliptically fibered Calabi–Yau’s as the volume of the fibers tends to zero (see also [4–7]). These types of degenerations relate to the conjectural picture of mirror symmetry put forth by Strominger–Yau–Zaslow [8], who postulate that mirror Calabi–Yau manifolds are given by dual torus fibrations over a real base with a singular affine structure. One major challenge when confronting this conjecture is the difficulty associated with constructing Lagrangian torus fibrations on a given Calabi–Yau. However, if one instead considers degenerations of Calabi–Yau’s, the fibration structure often becomes apparent in the limit.

Vafa’s extension of the mirror symmetry conjecture to include holomorphic bundles raises the question of how Yang–Mills connections behave under these degenerations [9]. For a general abelian fibered Calabi–Yau, Fukaya makes the following conjecture: Given a sequence of Yang–Mills connections on a family of Calabi–Yau metrics with collapsing fibers, there exists a rectifiable set in the base of real codimension at least 2, such that on all fibers away from this set, the connections have bounded curvature, and the restriction to each torus fiber converges to a flat connection [10, Conjecture 5.5]. In the context of SYZ mirror symmetry, a fiberwise flat connection will define a Lagrangian submanifold ${{\mathcal {L}}}$ in the mirror Calabi–Yau, and Fukaya further conjectures there exists a corresponding mirror sequence of Lagrangians converging to ${{\mathcal {L}}}$. In this paper we partially address the vector bundle portion of Fukaya’s conjecture, in the case of a fixed holomorphic *SU*(*n*) bundle over a *K*3 surface.

Our setup is as follows. Let $\pi :X\rightarrow {{\mathbb {P}}}^1$ be an elliptic *K*3 surface. Let $\omega _{{\mathbb {P}}^1}$ be a Kähler form on ${{\mathbb {P}}}^1$, and $\omega _X$ a Kähler form on *X*. Consider the family $\pi ^*\omega _{{\mathbb {P}}^1}+t^2 \omega _X$, which approaches the boundary of the Kähler cone as $t\rightarrow 0$, and let $\omega _t$ be the unique Ricci-flat metric in the class $[\pi ^*\omega _{{\mathbb {P}}^1}+t^2 \omega _X]$ given by Yau’s theorem [11]. Next, let $( V,{\bar{\partial }}_{\Xi })$ be a holomorphic *SU*(*n*) bundle over *X*, with a fixed metric $H_0$. Assume there exists a sequence $t_i\rightarrow 0$ such that the bundle *V* is stable with respect to $\omega _{t_i}$. By the theorem of Donaldson, Uhlenback–Yau [12, 13], there exists a corresponding sequence of connections $\Xi _i$ solving the Hermitian–Yang–Mills (HYM) equations:

$$\begin{aligned} F_{\Xi _i}\wedge \omega _{t_i}=0\qquad \mathrm{and}\qquad (F_{\Xi _i})^{0,2}=0. \end{aligned}$$

Furthermore, each $\Xi _i$ is complex gauge equivalent to $\Xi$, so they define the same holomorphic structure (see (3.1)). We now state our main result:

### Theorem 1.1

With the set-up as above:

1. There exists a finite subset $Z\subset {\mathbb {P}}^1$, such that for any $x\in {\mathbb {P}}^1\setminus Z$, if $E = \pi ^{-1}(x)$, then the restriction $\Xi _i\big |_{E}$ converges smoothly, along a subsequence and modulo unitary gauge transformations, to a flat connection on the fiber.
2. Furthermore, if the restriction $V|_E$ is isomorphic to a direct sum of line bundles $\oplus _{j=1}^n{\mathcal {O}}_E(q_j-0)$ for *n* distinct points $q_j\in E$, then the limiting flat connection is uniquely determined, and given by
  1.1 $$\begin{aligned} A_0=\frac{\pi }{\mathrm{Im}(\tau )} \left( \mathrm{diag}\{{\bar{q}}_1,\ldots ,{\bar{q}}_n\}dz-\mathrm{diag}\{q_1,\ldots ,q_n\}d{\bar{z}}\right) , \end{aligned}$$
  where *z* is the holomorphic coordinate on the fiber, and $\tau$ determines the complex structure. In this case, we also have the following convergence:
  $$\begin{aligned} ||\Xi _i|_E-A_0||_{L^2_1(E,H_0,g_0,A_0)}\rightarrow 0. \end{aligned}$$
  Here $g_0$ is a flat reference metric on *E*, and the flat connection $A_0$ is used to compute derivatives.

Note that in the second point above, no gauge transformations are needed for convergence. The first point follows from a bubbling argument. Our sequence of connections has bounded Yang–Mills energy, thus there can only be a finite number of bubbles, and we show away from these bubbles the curvature of $\Xi _i|_{E}$ must approach zero in the $C^0$ norm. This step closely follows two cases from Dostoglou–Salamon, from their proof of the Atiyah–Floer conjecture [14, 15]. The key difference here is that our Ricci flat metric $\omega _{t_i}$ is not a product metric, so we rely on certain convergence results for $\omega _{t_i}$.

It then follows that $\Xi _i|_{E}$ must approach some limiting flat connection, and the main contribution of this paper is the explicit identification of the limit, under the assumption that the restriction of the holomorphic bundle $V|_E$ is isomorphic to a direct sum of line bundles $\oplus _{j=1}^n{\mathcal {O}}_E(q_j-0)$. We use the observation that, because our sequence of connections $\Xi _i$ all define a fixed holomorphic structure, there exists a sequence of Hermitian endomorphisms $s_i$ satisfying $\Xi _i|_E=e^{s_i}( A_0)$ (where this action is defined by (3.1)). Although there is no hope of achieving $C^0$ control of $s_i$, we prove a gauge fixing result, and demonstrate that there exists a suitable normalization $s'_i$, defining the same connection, which in addition satisfies a uniform $C^0$ bound. This significant step is detailed in Theorem 5.1, which in particular hinges on a Poincaré inequality (5.1), where the explicit form of $V|_E$ is used. From here, convergence of $\Xi _i|_E$ to $A_0$ stated in Theorem 1.1 follows from standard theory.

Next, we turn to a specific geometric setup where Theorem 1.1 applies. Although this setup requires more assumptions, it has the benefit of producing explicit examples of bundles where the the limiting flat connection can be identified on a generic fiber. We now assume $\pi : X\rightarrow {{\mathbb {P}}}^1$ is a projective, elliptic *K*3 surface with a section $\sigma$ and singular fibers of type $I_1$ or *II*. Assume the restriction of *V* to a generic fiber is semi-stable and regular (see Sect. 3 for relevant definitions). Then by the work of Friedman–Morgan–Witten in [16], there exists a divisor $D_V\in |n\sigma ({\mathbb {P}}^1)+kl|$, called the *spectral cover* associated to *V*, where *l* denotes the effective divisor class of the fibers, and $k\in {\mathbb {Z}}$ satisfies $0\le k\le c_2(V)$. If $D_V$ is reduced and irreducible, then *V* is stable with respect to $\pi ^*[\omega _{{\mathbb {P}}^1}]+t^2[\omega _X]$ for any ample class $[\omega _X]$ on *X*, for $0<t^2\le (\frac{n^3}{4}c_2(V))^{-1}.$ Thus, for any sequence $t_i\rightarrow 0$ we can always find a corresponding sequence of HYM connections $\Xi _i$ on *V*. More importantly, the intersection of the spectral cover $D_V$ with a generic fiber *E* precisely picks out the points ${q_1,\ldots ,q_n}$ from (1.1), and so the limiting flat connection is uniquely determined away from the ramification points of $D_V$.

### Corollary 1.2

Assume $\pi : X\rightarrow {{\mathbb {P}}}^1$ is a projective, elliptic *K*3 surface with a section, with singular fibers of type $I_1$ or *II*. Let *V* be a holomorphic *SU*(*n*) bundle where the restriction to a generic fiber is semi-stable and regular, and assume the spectral cover $D_V$ is reduced and irreducible. Then for any sequence $t_i\rightarrow 0$, there exists a sequence of HYM connections $\Xi _i$ on *V* corresponding to $\omega _{t_i}$. Furthermore, away from a finite number of fibers, there exists a HYM connection $\Xi _0$ uniquely determined by $D_V$, satisfying $\Xi _0|_{E}=A_0$, where $A_0$ is defined via (1.1). Specifically, on a generic fiber *E* the points ${q_1,\ldots ,q_n}$ defining $A_0$ are given by

$$\begin{aligned} D_V\cap E=q_1+\cdots +q_n. \end{aligned}$$

On *E* we again have the convergence

$$\begin{aligned} ||\Xi _i|_E-\Xi _0|_E||_{L^2_1(E,H_0,g_0, A_0)}\rightarrow 0. \end{aligned}$$

The above setup is particularly attractive in that it allows for us to specify the limiting flat connection in a family that varies homomorphically in the base. Thus our result is a natural starting place to explore convergence in general, as opposed to only in the fiber direction.

Since adiabatic limits of Yang–Mills connections are fairly well studied, we now put our work in the context of previous results. Working on the product of two compact Riemann surfaces with trivial fibration $\pi :\Sigma _1\times \Sigma _2\rightarrow \Sigma _1$, J. Chen considers a family of metrics collapsing the fibers, and analyzes the convergence of a corresponding family of anti-self dual Yang–Mills connections [17, Theorem 4.10]. Assuming that $\Sigma _2$ has genus at least two, he proves that, modulo a sequence of gauge transformations and away from a bubbling set, the fiber component of the connection will converge continuously to a flat connection. Following this work, and using Fukaya’s gauge fixing theorem [18, Theorem 1.7], T. Nishinou improves upon Chen’s result, demonstrating smooth convergence away from a finite number of fibers and modulo gauge transformations, where the restriction of the limit to a fiber will be flat [19, Theorem 1.2]. This result requires the moduli space of flat connections over $\Sigma _2$ to be smooth and of expected dimension, with no reducible flat connections. The failure of such an assumption to hold over an elliptic curve is a major obstacle to extending the above results to elliptic fibrations.

In the case of *SU*(2) bundles over the product of elliptic curves, in [20] Nishinou is able to partially extend his above results, after utilizing his gauge fixing theorem from [21, Theorem 3.11]. In some ways, our Theorem 5.1 can be thought of as a generalization to higher rank of this gauge fixing theorem, although we have already assumed existence of a fixed holomorphic structure. In fact, this assumption serves as a major simplification throughout our paper, compared to the general case of a sequence of anti-self dual Yang–Mills connections considered in [14, 17–20]. The most notable simplification is that, because our sequence of connections $\Xi _i$ are all complex gauge equivalent to $\Xi$, we can bypass working with a sequence of holomorphic maps (which plays a role in [17, 19, 20]), as well as the more difficult type three bubbles of Dostoglou–Salamon [14, 15]. Additionally, this assumption allows us to prove the convergence in the second point of Theorem 1.1 directly, without relying on unitary gauge transformations.

Although our main result only applies to the restriction of $\Xi _i$ to each fiber, one may hope to demonstrate convergence on any compact set away from a finite number of fibers. Nishinou achieves this in [19] and [20], as his assumptions allow a Poincaré type inequality in a neighborhood of a fiber, even in the elliptic curve case (this follows from the estimate in Lemma 6.43 from [18]). This estimate implies that once $C^0$ control of the complexified gauge transformation is demonstrated on one fiber, it holds for nearby fibers. Unfortunately we are unable to extend Lemma 6.43 from [18] to our setting, as our Poincare inequality (Proposition 5.2) requires a normalization that only holds fiberwise. Another result in this direction is proven by Fu in [22], who considers a specific rank two bundle over the product of two elliptic curves which is given by a two sheeted spectral cover. He defines a reference metric which satisfies desired asymptotic behavior near the ramification points of the cover, and then demonstrates that the a sequence of HYM metrics will converge smoothly to this reference metric. Because in our setting we consider a spectral cover as well, one may hope to extend Fu’s result to the K3 surface case. Here, one major difficulty is the problem of constructing a reference metric near the singularities of the fibration. It is possible that the asymptotics of the metrics constructed in [23–25] may provide a clue, and we hope to investigate these types of constructions in future work.

Finally, we remark that after an earlier draft of this paper appeared, building on this work, the authors, along with Y. Zhang, were able to demonstrate convergence of $\Xi _i$ on compact sets away from a finite number of fibers, under the assumptions of Corollary 1.2. We direct the reader to [26] for details.

Our paper is organized as follows. In Sect. 2 we describe in detail semi-flat Kähler metrics on a K3 surface, which serve as a local model for our degenerating Ricci-flat metrics away from the singular fibers. Next in Sect. 3 we introduce the necessary background on holomorphic vector bundles over elliptic fibrations, and state some preliminary results. Our bubbling argument in described Sect. 4. We then turn to identifying the limiting flat connection, and prove our gauge fixing result is Sect. 5. In Sect. 6 we complete the proof of our main theorem, and demonstrate convergence of our connections.

## Semi-Flat Kähler Metrics

In this section, we review the construction of semi-flat Kähler metrics on a K3 surface. These metrics will not only describe the limiting behavior of the Ricci-flat metrics in dilated coordinates, and thus play a role in our bubbling argument, but they will also be useful for our understanding of the holomorphic structure of *V*. To begin, we introduce the notion of a special Kähler metric, which lives on the base of our elliptic fibration, and are a useful starting place to defining the semi-flat metric. We will closely follow the paper of Freed [27].

Let *B* be a Riemannian manifold of real dimension two. Assume *TB* admits a flat, torsion free connection $\nabla ^B$, which gives a covering of *B* by local affine coordinate charts. Furthermore, assume the coordinate transformations lie in $SL(2,{{\mathbb {R}}})$. Let $(x^1,x^2)$ be coordinates in a local chart, and let $\phi _{ij}dx^idx^j$ be a Hessian metric solving the real Monge-Ampere equation

2.1 $$\begin{aligned} \mathrm{det}(\phi _{ij})=1. \end{aligned}$$

Here, we use the notation $\phi _{ij}:=\frac{\partial ^2\phi }{\partial x^i\partial x^j}$ for a smooth function $\phi$ on *B*. We also denote $\frac{\partial \phi }{\partial x^i}$ by $\phi _i$.

Consider the locally defined 2-form $\omega _B= dx^1\wedge dx^2.$ Because $SL(2,{{\mathbb {R}}})= Sp(2,{{\mathbb {R}}})$, any matrix $A\in SL(2,{{\mathbb {R}}})$ preserves $\omega _B$, and thus $\omega _B$ is well defined on all of *B*. It defines both a natural symplectic form and a volume form. Furthermore, $\nabla ^B\omega _B=0$, and so $\nabla ^B$ is a symplectic connection. Taken together, $\omega _B$ and $\phi _{ij}$ define an almost complex structure *I* on *TB*, which in coordinates can be expressed by

$$\begin{aligned} I\left( \frac{\partial }{\partial x^k}\right) =-\phi _{k2}\frac{\partial }{\partial x^1}+\phi _{k1}\frac{\partial }{\partial x^2}, \end{aligned}$$

for $k=1,2$. One can show explicitly that Nijenhuis tensor of *I* vanishes and thus it is integrable.

### Definition 2.1

$(B,\omega _B, I)$ is special Kähler if it admits a real, flat, torsion-free, symplectic connection $\nabla ^B$ satisfying

$$\begin{aligned} d_{\nabla ^B} I=0. \end{aligned}$$

To see that *B* is special Kähler, note in affine coordinates the flat connection $\nabla ^B$ is simply given by *d*, and so

$$\begin{aligned} \frac{\partial }{\partial x^k}I^p{}_q -\frac{\partial }{\partial x^q}I^p{}_k =0, \end{aligned}$$

which follows because $\phi _{ij}$ is a Hessian metric.

Given the complex structure *I*, we can give holomorphic coordinate functions on *B*.

### Lemma 2.2

The functions

$$\begin{aligned} w=x^1+{\mathbf {i}}\phi _2\qquad \mathrm{and}\qquad \xi =-x^2+{\mathbf {i}}\phi _1. \end{aligned}$$

are holomorphic with respect to the complex structure *I*.

### Proof

Taking the exterior derivative gives $dw=(1+{\mathbf {i}}\phi _{12})dx^1+{\mathbf {i}}\phi _{22}dx^2$ and $d\xi ={\mathbf {i}}\phi _{11}dx^1-(1-{\mathbf {i}}\phi _{12})dx^2$. By our explicit representation of *I* it is easy to check that $I(dw)={\mathbf {i}}dw$ and $I(d\xi )={\mathbf {i}}d\xi$. $\square$

For the remainder of the paper we choose *w* as our holomorphic coordinate on the base. We would like to better understand the holomorphic vector field $\frac{\partial }{\partial w}$. First, note that the coordinate transformation $T(x^1, x^2)\rightarrow (w, {\bar{w}})$ has pushforward matrix

$$\begin{aligned} T_*=\left( \begin{array}{cc} 1+{\mathbf {i}}\phi _{12} &{}{\mathbf {i}}\phi _{22}\\ 1-{\mathbf {i}}\phi _{12}&{}-{\mathbf {i}}\phi _{22}\end{array} \right) \qquad \mathrm{and}\qquad T_*^{-1}=\frac{1}{2}\left( \begin{array}{cc} 1 &{}1\\ -{\mathbf {i}}\frac{\phi _{11}}{1+{\mathbf {i}}\phi _{12}}&{}{\mathbf {i}}\frac{\phi _{11}}{1-{\mathbf {i}}\phi _{12}}\end{array} \right) , \end{aligned}$$

where we have used (2.1). We now compute the partial derivative

2.2 $$\begin{aligned} \frac{\partial \xi }{\partial w}= & {} \frac{\partial \xi }{\partial x^1}\frac{\partial x^1}{\partial w}+\frac{\partial \xi }{\partial x^2}\frac{\partial x^2}{\partial w}\nonumber \\= & {} ({\mathbf {i}}\phi _{11})\left( \frac{1}{2}\right) +(-1+{\mathbf {i}}\phi _{12})\left( \frac{-{\mathbf {i}}}{2}\frac{\phi _{11}}{1+{\mathbf {i}}\phi _{12}}\right) \nonumber \\= & {} \frac{{\mathbf {i}}}{2}\phi _{11}\left( 1+\frac{1-{\mathbf {i}}\phi _{12}}{1+{\mathbf {i}}\phi _{12}}\right) ={\mathbf {i}}\frac{\phi _{11}}{1+{\mathbf {i}}\phi _{12}}={\mathbf {i}}\frac{1-{\mathbf {i}}\phi _{12}}{\phi _{22}}, \end{aligned}$$

where the last equality follows from (2.1). For simplicity we will use the notation $\tau :=\frac{\partial \xi }{\partial w},$ as $\tau$ will define the complex structure of our elliptic fibers. Then, by the explicit formula from Freed [27, Equation (1.12)], we see

$$\begin{aligned} \frac{\partial }{\partial w}=\frac{1}{2}\left( \frac{\partial }{\partial x^1}-\tau \frac{\partial }{\partial x^2}\right) . \end{aligned}$$

Next we construct a hyper-Kähler structure on *TB*. Quotienting out *TB* by a lattice $\Lambda$ will give a local model for our elliptic fibration *X* away from the singular fibers. To begin, consider the following extension of our Hessian metric to *TB*:

$$\begin{aligned} g=\phi _{ij}\left( {dx^idx^j}+dy^i dy^j\right) . \end{aligned}$$

With this metric we define three complex structures which make up a hyper-Kähler triple.

By a slight abuse of notation, let *I* denote the complex structure on *TB* induced from the complex structure *I* on the base. In particular *I* can be expressed as

$$\begin{aligned} I\left( \frac{\partial }{\partial x^k}\right) =-\phi _{k2}\frac{\partial }{\partial x^1}+\phi _{k1}\frac{\partial }{\partial x^2}\qquad \mathrm{and}{\qquad }I\left( \frac{\partial }{\partial y^k}\right) =\phi _{k2}\frac{\partial }{\partial y^1}-\phi _{k1}\frac{\partial }{\partial y^2} \end{aligned}$$

for $k=1,2$. The corresponding Kähler form is given by

2.3 $$\begin{aligned} \omega _{I}= {dx^1\wedge dx^2}-dy^1\wedge dy^2. \end{aligned}$$

In this complex structure the fibers are holomorphic subvarieties.

Next, we consider a complex structures *J* where the fibers are special Lagrangian, defined by

$$\begin{aligned} J \left( \frac{\partial }{\partial x^k}\right) = \frac{\partial }{\partial y^k}\qquad \mathrm{and}{\qquad }J \left( \frac{\partial }{\partial y^k}\right) = \frac{\partial }{\partial x^k}. \end{aligned}$$

Using the metric *g* the corresponding Kähler form is

$$\begin{aligned} \omega _J=\phi _{ij} dx^i\wedge dy^j. \end{aligned}$$

Finally, one can define the complex structure $K=JI$, which together with *g* gives the Kähler form

$$\begin{aligned} \omega _K=dx^1\wedge dy^2-dx^2\wedge dy^1. \end{aligned}$$

It is easy to see that $\omega _{I }$, $\omega _J$ and $\omega _K$ are closed, and by a lemma of Hitchin [28] it follows that *I*, *J*, and *K* are integrable complex structures. In the standard fashion we can construct the following top dimensional holomorphic forms:

$$\begin{aligned}&\Omega _I=\omega _K+{\mathbf {i}}\,\omega _J \\&\Omega _{J}=\omega _{I}+{\mathbf {i}}\,\omega _K \\&\Omega _{K}=\omega _{I}+{\mathbf {i}}\,\omega _J, \end{aligned}$$

giving hyper-Käher triple. Note the metric and complex structures defined above are invariant under translation in the $y-$coordinates. Thus, if $\Lambda$ is the standard lattice $\langle 1,1\rangle$, the entire setup will descend to the elliptically fibered manifold $TB/\Lambda$.

We now construct complex coordinates on $TB/\Lambda$. We have a complex coordinate *w* on the base *B*, and in the fiber direction we define

2.4 $$\begin{aligned} z=\tau y^1+y^2. \end{aligned}$$

Here, $\tau$ represents the complex period of the elliptic curve, and is defined above in (2.2). By definition $\tau :=\frac{\partial \xi }{\partial w}$, and since $\xi$ is a holomorphic function, $\tau$ is holomorphic in *w* as well. This leads to the following:

### Lemma 2.3

The coordinates (*w*, *z*) are holomorphic coordinates on $TB/\Lambda$ with respect to *I*.

### Proof

Lemma 2.2 shows that *w* is holomorphic, and so it remains to be seen that $dz(V)=0$ for all vector fields *V* of type (0, 1). Taking the exterior derivative of *z* gives

2.5 $$\begin{aligned} dz=\tau dy^1+y^1\frac{\partial \tau }{\partial w}dw+dy^2, \end{aligned}$$

where we used $\tau$ is holomorphic. At first glance, the term $y^1\frac{\partial \tau }{\partial w}dw$ may seem out of place, however, it is important to remember that unless $\tau$ is constant, our local picture is not the cartesian product of the base with an elliptic curve, and so this term is expected.

Consider the vector field

$$\begin{aligned} \frac{\partial }{\partial {\bar{z}}}=\frac{1}{2}\left( \frac{\partial }{\partial y^1}-\frac{{\mathbf {i}}\phi _{11}}{1+{\mathbf {i}}\phi _{12}}\frac{\partial }{\partial y^2}\right) . \end{aligned}$$

From the explicit form of *dz*, we see that in order for $\frac{\partial }{\partial {\bar{z}}}$ to be anti-holomorphic, it needs to be killed by the form *dw* on the base.

Using the definition of *I*, and the fact that $\mathrm{det}(\phi _{ij})=1$, we compute

$$\begin{aligned} I\left( \frac{\partial }{\partial {\bar{z}}}\right)= & {} \frac{1}{2}\left( \phi _{12}-\frac{{\mathbf {i}}\phi _{11}\phi _{22}}{1+{\mathbf {i}}\phi _{12}}\right) \frac{\partial }{\partial y^1}+\frac{1}{2}\left( -\phi _{11}+\frac{{\mathbf {i}}\phi _{11}\phi _{12}}{1+{\mathbf {i}}\phi _{12}} \right) \frac{\partial }{\partial y^2}\\= & {} \frac{1}{2}\left( -{\mathbf {i}}\right) \frac{\partial }{\partial y^1}+\frac{1}{2}\left( -\frac{\phi _{11}}{1+{\mathbf {i}}\phi _{12}} \right) \frac{\partial }{\partial y^2}=-{\mathbf {i}}\frac{\partial }{\partial {\bar{z}}}, \end{aligned}$$

which demonstrates this vector field is of type (0, 1). By Lemma 2.2 it follows that $dw\left( \frac{\partial }{\partial {\bar{z}}}\right) =0$. Additionally, we have

$$\begin{aligned} (\tau dy^1+dy^2)\left( \frac{\partial }{\partial {\bar{z}}}\right) =\frac{1}{2}(\tau -\tau )=0. \end{aligned}$$

So $dz(\frac{\partial }{\partial {\bar{z}}})=0$. Since $\frac{\partial }{\partial {\bar{w}}}$ and $\frac{\partial }{\partial {\bar{z}}}$ span all local (0, 1) vector fields, we conclude that (*w*, *z*) are holomorphic coordinates. $\square$

We conclude this section with a more detailed discussion of how the Ricci-flat Kähler metrics behave in the limit. We recall our setup from the introduction. Let $\pi :X\rightarrow {{\mathbb {P}}}^1$ be an elliptic *K*3 surface, and denote by $Z_\pi$ the image of the singular fibers. Let $\omega _{{\mathbb {P}}^1}$ be the Fubini-Study metric on on ${{\mathbb {P}}}^1$, and $\omega _X$ a Kähler form on *X*. Let $\omega _t$ be the unique Ricci-flat metric in the class $[\pi ^*\omega _{{\mathbb {P}}^1}+t^2 \omega _X]$. The convergence we need is local, so we fix a small, simply connected open set $U\subset {\mathbb {P}}^1$ away from $Z_\pi$, and define $X_U:=\pi ^{-1}(U)$. On *U* we can consider the Kähler form $\omega _B$ along with the Hessian metric $\phi _{ij}$, which on this small open set is equivalent to $\omega _{{\mathbb {P}}^1}$. On $X_U$ we have the fixed background metric $\omega _X$, but we also have the Kähler form $\omega _I$ as defined above, called the *semi-flat metric*, and we denote it by $\omega _I=:\omega _{SF}$ for emphasis.

We will need the following uniform equivalence result. By [2, Lemma 4.1], there exists a constant *C* so that for *t* small enough

2.6 $$\begin{aligned} C^{-1}\left( \pi ^*\omega _{{\mathbb {P}}^1}+t^2\,\omega _X\right) \le \omega _t\le C\left( \pi ^*\omega _{{\mathbb {P}}^1}+t^2\,\omega _X\right) . \end{aligned}$$

We also need a result that demonstrates how $\omega _t$ degenerates. Consider the projection $p:U\times {\mathbb {C}}\rightarrow (U\times {\mathbb {C}})/\Lambda =:X_U$, and the coordinate transformation $L_t: U\times {\mathbb {C}}\rightarrow U\times {\mathbb {C}}$ defined by

2.7 $$\begin{aligned} L_t(x,y)=(x,\frac{y}{ t}). \end{aligned}$$

This coordinate transformation is a dilatation, designed so that the size of the fibers of $\pi$ with respect to the metric $L_t^*p^*\omega _t$ are fixed. We will use the following result of Hein-Tosatti from [4, Proof of Theorem 1.1], which demonstrates how the semi-flat metric serves as a model for the limiting behavior of $\omega _t$.

### Proposition 2.4

(Hein-Tosatti [4]) There exists a constant *C* so that for *t* small enough

$$\begin{aligned} C^{-1}p^*\left( \pi ^*\omega _{B}+\omega _{SF}\right) \le L_t^*p^*\omega _t\le Cp^*\left( \pi ^*\omega _{B}+\omega _{SF}\right) . \end{aligned}$$

This estimate also appears in [7], with the extra assumption that *X* is projective. For more details on convergence results, we direct the reader to [2, 3].

## Holomorphic Bundles over Elliptic Manifolds

In this section, we provide the necessary background on holomorphic vector bundles, including the relevant notions of stability needed to construct our sequence of HYM connections. We also introduce the construction of a spectral cover associated to *V*, following Friedman–Morgan–Witten [16] (see also [29–31]), and conclude the section with the construction of $\Xi _0$ used in Corollary 1.2.

To begin, suppose $(X,\omega )$ is a compact Kähler manifold of complex dimension *m*. Let $( V, {\bar{\partial }}_{\Xi })$ be a holomorphic bundle over *X*. For any Hermitian metric $H_0$ on *V*, there exists a unique connection, called the Chern connection, compatible with both the metric and the holomorphic structure, which we denote by $\Xi$. The *degree* of *V* is defined by the following integral:

$$\begin{aligned} \mathrm{deg}( V,\omega )={\mathbf {i}}\int _X\mathrm{Tr}(F_{\Xi })\wedge \omega ^{m-1}. \end{aligned}$$

Given two metrics on *V*, the curvatures of the two corresponding Chern connections will differ by a $\partial {\bar{\partial }}$-exact term, demonstrating that the degree is independent of a choice of metric. Furthermore, the degree does not depend on the representative of the Kähler class $[\omega ]$. However, for $m>1$, changing the class may change the degree.

### Definition 3.1

$( V,\omega )$ is *stable* if, for all proper, torsion-free subsheaves ${\mathcal {F}}\subset E$,

$$\begin{aligned} \frac{\mathrm{deg}({\mathcal {F}},\omega )}{\mathrm{rk}({\mathcal {F}})}<\frac{\mathrm{deg}( V,\omega )}{\mathrm{rk}( V)}. \end{aligned}$$

$( V,\omega )$ is *semi-stable* if the above expression holds with a weak inequality.

Note that if ${\mathcal {F}}$ is not locally free, its degree is defined by computing the degree of $\mathrm{det}({\mathcal {F}})$, which is always a line bundle.

On any complex manifold, the space of one forms decomposes into the eigenspaces for $\pm {\mathbf {i}}$ with respect to the complex structure. This allows us to write the any connection $\Xi$ as $\Xi =\Xi ^{1,0}+\Xi ^{0,1}$. Using this decomposition, one can define an action of the complexified gauge group on the space of connections. Specifically, if $\sigma \in GL( V)$, then

3.1 $$\begin{aligned} \sigma (\Xi )=\sigma ^*{}^{-1} \Xi ^{1,0} \sigma ^*+\sigma ^*{}^{-1}\partial \sigma ^*+\sigma \Xi ^{0,1} \sigma ^{-1}-{\bar{\partial }}\sigma \sigma ^{-1}, \end{aligned}$$

Note that if $\sigma$ is in fact unitary, the above action reduces to the standard action of the unitary gauge group. In this case, we use the standard notation $u^*\Xi$ for the unitary action.

We now turn to the HYM equations on a general Kähler manifold:

3.2 $$\begin{aligned} {\mathbf {i}}F_{\Xi }\wedge \omega ^{m-1}=\frac{m\,\mathrm{deg}(V,\omega )}{\mathrm{rk(V)}\mathrm{Vol}(X)} Id_V\,{\omega ^m}\qquad \mathrm{and}\qquad (F_{\Xi })^{0,2}=0. \end{aligned}$$

For any metric *g* on *X* and connection $\Xi$ on *V*, the Yang–Mills energy is defined by the following integral

$$\begin{aligned} {\mathcal {Y}}{\mathcal {M}}(\Xi ,g) := \int _X|F_{\Xi }|^2_{H_0, g}\,dV_g. \end{aligned}$$

Critical points of this energy functional are called Yang–Mills connections, and one can check using the Kähler identities that HYM connections are are special class of Yang–Mills connections which are compatible with the complex structure on *X*.

Note that second equation in (3.2) stipulates that $\Xi$ is compatible with the holomorphic structure on *V*. By definition this second equation is satisfied by the Chern connection $\Xi$, and one can check this compatibility is preserved along the action (3.1). This leads to the following question: Given a holomorphic vector bundle with fixed metric $H_0$, does there exist a solution ${\hat{\Xi }}$ to (3.2) in the orbit of (3.1)? A definitive answer to this question was given by Donaldson, Uhlenbeck–Yau, in the following fundamental result.

### Theorem 3.2

(Donaldson [12], Uhlenbeck–Yau [13]) A holomorphic bundle *V* over $(X,\omega )$ admits a unique Hermitian–Yang–Mills connection in the complex gauge orbit of the Chern connection if and only if it is stable.

In fact, one can prove that if ${\hat{\Xi }}$ is the unique Hermitian–Yang–Mills connection, it can be expressed as ${\hat{\Xi }} =e^s (\Xi )$, where *s* is a trace free Hermitian endomorphism of *V*.

Given this background, we return to our setup. Let $$\pi :X\rightarrow {\mathbb {P}}^1$$ be an elliptically fibered *K*3 surface, and let $$\omega _{t}$$ be the unique Ricci-flat Kähler metric in the class $$[\pi ^*\omega _{{\mathbb {P}}^1}+t^2\,\omega _X]$$. Assume $$( V,{\bar{\partial }}_{\Xi })$$ is a holomorphic *SU*(*n*) bundle over *X*. This implies the curvature $$F_{\Xi }$$ is trace free, and so $$\mathrm{deg}( V, \omega _{t})=0$$ for all *t*. Furthermore, assume that $$( V,{\bar{\partial }}_{\Xi })$$ is stable with respect to $$\omega _{t_i}$$ for some sequence $$t_i\rightarrow 0$$. Let $$ g_{t_i}$$ be the Kähler metrics associated to $$\omega _{t_i}$$. By the theorem of Donaldson–Uhlenbeck–Yau there exists a corresponding sequence of HYM connections $$\Xi _i$$ on *V*. Note that in our particular setting, the HYM equations take the simpler form

$$\begin{aligned} F_{\Xi }\wedge \omega =0\qquad \mathrm{and}\qquad (F_{\Xi })^{0,2}=0. \end{aligned}$$

We will need the following Lemma, which states that the Yang–Mills energy of a HYM connection is a topological invariant. This result is standard, and can be found, for instance, in [32]. We include a proof for the reader’s convenience.

### Lemma 3.3

The Yang–Mills energy of $\Xi _i$ with respect to the metric $g_{t_i}$, is fixed, i.e.

$$\begin{aligned} {\mathcal {Y}}{\mathcal {M}}(\Xi _i,g_{t_i})={\mathcal {Y}}{\mathcal {M}}(\Xi , g_{t_0}). \end{aligned}$$

### Proof

Let ${\mathbf {i}}\Lambda _\omega$ denote the adjoint of wedging with the Kähler form $\omega$. Then, the equation $F_\Xi \wedge \omega =0$ can be equivalently expressed as ${\mathbf {i}}\Lambda _\omega F_\Xi =0$. Equality (4.4.5) in [33] shows that for any complex surface *X* one has

$$\begin{aligned} \int _X\mathrm{Tr}(F_{\Xi _i}\wedge F_{\Xi _i})=||F_{\Xi _i}||^2_{L^2(H_0, g_{t_i})}-||{\mathbf {i}}\Lambda _{\omega _{t_i}}F_{\Xi _t}||^2_{L^2(H_0,g_{t_i})}. \end{aligned}$$

Since $F_{\Xi _i}$ is HYM with respect to $g_{t_i}$, the right most term vanishes, and so

$$\begin{aligned} {\mathcal {Y}}{\mathcal {M}}(\Xi _i, g_{t_i})=\int _X\mathrm{Tr}(F_{\Xi _i}\wedge F_{\Xi _i}). \end{aligned}$$

The right-hand side above yields a topological invariant $[c_2( V)-\frac{1}{2}c_1^2( V)]\cup X$, and is thus independent of *i*, proving the lemma. $\square$

We now review the Friedman–Morgan–Witten construction of stable holomorphic bundles on elliptic fibrations with sections, and is needed for Corollary 1.2. We begin by looking at a single fiber. Let *E* be an elliptic curve, and $0\in E$ the identity of the group law. Denote the trivial line bundle by ${{\mathcal {O}}}$, and given a point $q\in E$, let ${\mathcal {O}}_E(q-0)$ be the line bundle associated to the divisor $q-0$. We also define a sequence of rank *r* bundles (denoted ${{\mathcal {I}}}_r$) inductively, with ${{\mathcal {I}}}_1={{\mathcal {O}}}$ and ${{\mathcal {I}}}_r$ the unique nontrivial extension of ${{\mathcal {I}}}_{r-1}$ by ${{\mathcal {O}}}$. Recall the following theorem of Atiyah (Theorem 5 from [34]):

### Theorem 3.4

(Atiyah [34]) Any semi-stable, degree zero bundle *V* over *E* is isomorphic to a direct sum of bundles of the form ${\mathcal {O}}_E(q-0)\otimes {{\mathcal {I}}}_r$, i.e.

$$\begin{aligned} V\cong \bigoplus _{j=1}^\ell {\mathcal {O}}_E(q_j-0)\otimes {{\mathcal {I}}}_{r_j}. \end{aligned}$$

### Definition 3.5

A semi-stable bundle is called regular if in the above direct sum $q_i\ne q_j$ for $i\ne j$.

Note that bundles of the form ${\mathcal {O}}_E(q_j-0)\otimes {{\mathcal {I}}}_{r_j}$ do not admit flat connections unless $r_j=1$. However, we can instead replace ${\mathcal {O}}_E(q_j-0)\otimes {{\mathcal {I}}}_{r_j}$ with its Seshadri filtration ${\mathcal {O}}_E(q_j-0)^{\oplus {r_j}}$, and define all bundles with the same Seshadri filtration to be ${{\mathcal {S}}}$-equivalent. We then see the ${{\mathcal {S}}}$-equivalence class of an *SU*(*n*) bundle is determined by *n* points $q_1,\ldots , q_n$ (counted with multiplicities) satisfying $q_1+\cdots +q_n=0$.

Thus, we can describe the moduli space of ${{\mathcal {S}}}$-equivalence classes of *SU*(*n*) bundles as follows. Let $W:=H^0(E,{{\mathcal {O}}}(n0))$ be the space of meromorphic functions $\phi$ that have a pole of at most order *n* at 0, with no other poles. By Abel’s Theorem $\phi$ must have *n* zeros satisfying $q_1+\cdots +q_n=0$. If $\phi$ has a pole of order less than *n* at 0, we interpret this as some of the $q_i$ are 0. The zeros of $\phi$ are preserved under multiplication by an element of ${{\mathbb {C}}}^*$, and so the moduli space is ${{\mathbb {P}}}W\cong {{\mathbb {P}}}^{n-1}$.

Next consider a projective elliptic fibration $\pi : X\rightarrow {{\mathbb {P}}}^1$, with singular fibers of type $I_1$ or *II* (this extra assumption gives that *X* coincides with its Weierstrass model). Over each generic point *x* in the base there is an elliptic curve $E_x:=\pi ^{-1}(x)$ and a moduli space ${{\mathbb {P}}}^{n-1}_x$ of *SU*(*n*) bundles. Friedman–Morgan–Witten demonstrate that the projective spaces glue together to form a ${{\mathbb {P}}}^{n-1}$ bundle over the base, which we denote by ${{\mathcal {W}}}$. A holomorphic *SU*(*n*) bundle *V* over *X* which restricts to a semi-stable bundle on each fiber determines a section *s* of ${{\mathcal {W}}}$, which in turn defines a divsor $D_V\subset X$. Specifically, each point *x* in the base determines *n* points in $E_x$, thus $D_V$ is an n-fold ramified cover of ${{\mathbb {P}}}^1$. More precisely, in Sect. 4 of [16] it is demonstrated:

### Theorem 3.6

(Friedman–Morgan–Witten [16]) Let $\pi : X\rightarrow {{\mathbb {P}}}^1$ be an elliptic fibration with a section $\sigma$, with singular fibers of type $I_1$ or *II*. Let *V* be a holomorphic bundle of rank *n* over *X*. Assume that the restriction of *V* to a generic fiber of $\pi$ is semi-stable and regular. Then, there exists a divisor

$$\begin{aligned} D_V\in |n\sigma ({\mathbb {P}}^1)+kl|, \end{aligned}$$

called the spectral cover associated to *V*, where *l* denotes the effective divisor class of the fibers of $\pi$, $k\in {\mathbb {Z}}$ satisfies $0\le k\le c_2(V)$. For a generic $x\in {\mathbb {P}}^1\backslash Z_\pi$, if $V|_{E_x}\cong \bigoplus _{j=1}^\ell {\mathcal {O}}_E(q_j-0)\otimes {{\mathcal {I}}}_{r_j}$, then

$$\begin{aligned} D_V\cap E_x=\sum _{j=1}^\ell r_jq_j\in |n\sigma (x)|. \end{aligned}$$

If *V* admits a spectral cover $D_V$ which is reduced and irreducible, then $D_V$ has a finite number of ramification points. Let $Z_D$ denote the image of these ramification point under $\pi$. Then, for any $x\in {\mathbb {P}}^1\backslash (Z_\pi \cup Z_D)$, we have $D_V\cap E_x=\sum _{j=1}^n q_j$ with all $q_j$ distinct, and thus

$$\begin{aligned} V|_{E_x}\cong {\mathcal {O}}_E(q_1-0)\oplus \cdots \oplus {\mathcal {O}}_E(q_n-0). \end{aligned}$$

This verifies the holomorphic structure assumption on $V|_{E_x}$ in Theorem 1.1. Furthermore the points $q_j$ vary holomorphically in *x*. The condition that $D_V$ be reduced and irreducible also guarantees that the bundle *V* is stable with respect to $\omega _t$ for small *t*. This can be used to construct many examples of connections $\Xi _i$ that satisfy the assumptions of our main theorem.

### Theorem 3.7

(Theorem 7.4 in [16]) If the spectral cover $D_V$ constructed above is reduced and irreducible, then *V* is stable with respect to $\pi ^*[\omega _{{\mathbb {P}}^1}]+t^2[\omega _X]$ for any ample class $[\omega _X]$ on *X*, for all $0<t^2\le (\frac{n^3}{4}c_2(V))^{-1}.$

We end this section with the construction of $\Xi _0$ from Corollary 1.2, which is a local *HYM* connection that determines the limit $A_0=\Xi _0|_E$ on each fiber. Although the limiting connection $A_0$ is expressed in holomorphic coordinates in (1.1), here we find it easier to work with our coordinates $(x^1,x^2, y^1, y^2)$ from the previous section. Both viewpoints are, of course, equivalent.

Consider $X_U:=\pi ^{-1}(U)$ for some simply connected $U\subset P^1\backslash (Z_\pi \cup Z_D)$. As a first step, we consider the case where *V* has rank one. Since it has degree zero, the bundle *V* is topologically trivial along each fiber and thus topologically trivial on $X_U$. We equip *V* with a trivial metric $H_0$, and fix a unitary frame. For a fiber $E_x$ we have assumed the restriction $V|_{E_x}\cong {\mathcal {O}}_E(q -0)$, with *q* varying holomorphically in the base. We decompose *q* as follows

3.3 $$\begin{aligned} q(x^1,x^2)=\theta _1(x^1,x^2)-\tau \theta _2(x^1,x^2). \end{aligned}$$

Recall $E_x$ is determined by the quotient $T_xB/\Lambda$, and so the point *q* can be lifted to a point $\tilde{q}$ in $T_xB\cong {{\mathbb {C}}}$. Now, if $\tau (x^1, x^2)$ gives the complex structure on $E_x$, we can define the holomorphic structure on *V* by

$$\begin{aligned} {\bar{\partial }}_q:={\bar{\partial }}-\frac{\pi \,\tilde{q}}{\mathrm{Im}(\tau )}\,d{\bar{z}}, \end{aligned}$$

where *z* is the complex coordiante defined in (2.4). At the end of the section we will demonstrate that the connections we construct are independent of the lift from *q* to $\tilde{q}$, and therefore well defined.

Given the above holomorphic structure, and using that $H_0$ is the trivial metric, the Chern connection can be computed as

$$\begin{aligned} \Xi _0=2\pi {\mathbf {i}}\,\left( \theta _1dy^1+\theta _2dy^2\right) . \end{aligned}$$

Since each $\theta _j$ only depends on the base coordinates, $\Xi _0|_{E_x}$ is flat on each fiber. Using (3.3), one can check that the expression for $\Xi _0|_{E_x}=:A_0$ is equivalent to (1.1) in the statement of the main theorem. The holonomy around each period in $E_x$ is given by $e^{2\pi {\mathbf {i}}\,\theta _1}$ and $e^{2\pi {\mathbf {i}}\,\theta _2}$, respectively.

### Proposition 3.8

The connection $\Xi _0$ is HYM with respect to all three complex structures *I*, *J*, and *K*, on $X_U$.

### Proof

As a first step we show

3.4 $$\begin{aligned} \phi ^{ij}\frac{\partial }{\partial x^i}\theta _j =0 \end{aligned}$$

and

3.5 $$\begin{aligned} \frac{\partial }{\partial x^i}\theta _j=\frac{\partial }{\partial x^j}\theta _i. \end{aligned}$$

To see this, because both $\tau$ and *q* are holomorphic in the base, one can compute

$$\begin{aligned} 0=\frac{\partial }{\partial {\bar{w}}}q= & {} \frac{1}{2}\left( \frac{\partial }{\partial x^1}-{\bar{\tau }}\frac{\partial }{\partial x^2}\right) (\theta _1-\tau \theta _2)\\= & {} \frac{1}{2}\left( \frac{\partial }{\partial x^1}\theta _1-{\bar{\tau }}\frac{\partial }{\partial x^2}\theta _1-\tau \frac{\partial }{\partial x^1}\theta _2+|\tau |^2\frac{\partial }{\partial x^2}\theta _2\right) . \end{aligned}$$

Now, using (2.2), the norm of $\tau$ is given by

$$\begin{aligned} |\tau |^2=\frac{(1-{\mathbf {i}}\phi _{12})(1+{\mathbf {i}}\phi _{21})}{\phi _{22}^2}=\frac{1+\phi _{12}^2}{\phi _{22}^2}=\frac{\phi _{11}}{\phi _{22}}, \end{aligned}$$

where for the last equality we used det$(\phi _{ij})=1$. Thus

$$\begin{aligned} 0=\frac{1}{2\phi _{22}}\left( \phi _{22}\frac{\partial }{\partial x^1}\theta _1+({\mathbf {i}}-\phi _{12})\frac{\partial }{\partial x^2}\theta _1-({\mathbf {i}}+\phi _{12})\frac{\partial }{\partial x^1}\theta _2+\phi _{11}\frac{\partial }{\partial x^2}\theta _2\right) . \end{aligned}$$

Since both the real and imaginary parts vanish, (3.4) and (3.5) follow. In particular (3.5) allows us to simplify our notation and denote $\frac{\partial }{\partial x^i}{\theta _j}$ as $\theta _{ij}$, where the indices commute.

The curvature of $\Xi _0$ is now given by

$$\begin{aligned} F_{\Xi _0}={2\pi {\mathbf {i}}}\,\theta _{ij} dx^i\wedge dy^j. \end{aligned}$$

Right away it follows that $F_{\Xi _0}\wedge \omega _{I}=0$ for all *t*. Furthermore, (3.4) implies $F_{\Xi _0}\wedge \omega _J=0$ and (3.5) gives $F_{\Xi _0}\wedge \omega _{K}=0$. Thus $\Xi _0$ is a holomorphic and HYM with respect to each complex structure. $\square$

We now turn to the general case. Assume $V|_{E_x}\cong \oplus _{j=1}^n{\mathcal {O}}_E(q_j-0),$ with each $q_j$ is distinct. As before write $q_j=\theta _1^j-\tau \theta _2^j$, and construct diagonal matrices $\Theta _1$ and $\Theta _2$ with eigenvalues $\theta _1^j$ and $\theta _2^j$, respectively. Consider the connection

3.6 $$\begin{aligned} {\Xi _0}=2\pi {\mathbf {i}}\left( \Theta _1dy^1+\Theta _2 dy^2\right) , \end{aligned}$$

Its curvature is given by

$$\begin{aligned} F_{\Xi _0}={2\pi {\mathbf {i}}}\,\Theta _{ij}dx^i\wedge dy^j. \end{aligned}$$

It is clear that $F_{\Xi _0}|_{E_x}=0$ for every fiber in $X_U$. Furthermore, by Proposition 3.8 we have

$$\begin{aligned} F_{\Xi _0}\wedge \omega _{I}=F_{\Xi _0}\wedge \omega _J=F_{\Xi _0}\wedge \omega _K=0. \end{aligned}$$

Thus $\Xi _0$ is a local HYM connection with respect to each complex structure, although we only focus on *I* in this paper.

Finally, we demonstrate that the lift of each point $q_i$ in $E_x$ to ${\mathbb {C}}$ is well defined. Recall that in the coordinates $(y^1,y^2)$, our lattice $\Lambda$ is the standard lattice given by $\langle 1,1\rangle$. Now, suppose we have another connection $\Psi$ satisfying

$$\begin{aligned} {\Xi _0}-\Psi =2\pi {\mathbf {i}}\left( M_1dy^1+M_2 dy^2\right) , \end{aligned}$$

where $M_1=$diag$(\alpha _1,\ldots ,\alpha _n)$ and $M_2=$diag$(\beta _1,\ldots ,\beta _n)$ are both diagonal matrices of integers, which means both $\Xi _0$ and $\Psi$ define the same points on $E_x$. If *u* is the gauge transformation given by

$$\begin{aligned} u=\mathrm{diag}(e^{2\pi {\mathbf {i}}(\alpha _1 y^1+\beta _1 y^2)},\ldots ,e^{2\pi {\mathbf {i}}(\alpha _n y^1+\beta _n y^2)}), \end{aligned}$$

we have ${\Xi _0}- \Psi =-duu^{-1}$. Furthermore because all the $\alpha _i$ and $\beta _i$ are integers, *u* descends to a smooth gauge transformation on the torus fibers, and thus the connections are gauge equivalent on $X_U$. Finally, since the points $q_i$ add up to 0 in the group law on $E_x$, we can find a lift to ${\mathbb {C}}$ where the points still add to 0, and $\Xi _0$ will be trace free.

## Bubbling

We now present our bubbling argument, following the first two cases from [14]. Consider a sequence of Hermitian–Yang–Mills connections $\Xi _i$, corresponding to $\omega _{t_i}\in [\omega _{{\mathbb {P}}^1}+t^2_{i}\omega _X]$ as $t_i\rightarrow 0$. Currently our argument depends on the sequence of connections we choose, although one can hope that with further analysis the set where bubbles occur can be uniquely identified by $(V,\partial _{\Xi })$.

Choose a compact set $K\subset {\mathbb {P}}^1\backslash Z_\pi$, where as before $Z_\pi$ is the image of the singular fibers under $\pi$. In a neighborhood *U* of any point $x\in K$, we can choose affine coordinates $(x^1,x^2)$ where *x* is at the origin, and coordinates $(x^1,x^2,y^1,y^2)$ on $X_U:=\pi ^{-1}(U)$. The curvature of $\Xi _i$ can be decomposed as

$$\begin{aligned} F_{\Xi _i} = F_{B_i} + F_{A_i} + \kappa _i, \end{aligned}$$

where $F_{B_i}$ and $F_{A_i}$ denote the base and fiber directions of the curvature, and $\kappa _i$ denotes the mixed terms. For each $x\in K$ we define the quantity

$$\begin{aligned} m_i(x):=||F_{B_i}||_{L^\infty (E_x,H_0,g_{X})} + \frac{1}{t_i^2}||F_{A_i}||_{L^\infty (E_x,H_0,g_{X})}+||\kappa _i||^2_{L^\infty (E_x, H_0, g_{X})}, \end{aligned}$$

where $g_{X}$ is the metric associated to the fixed Kähler form $\omega _{X}$.

### Proposition 4.1

There exists a finite number of points $\{p_1,\ldots ,p_\ell \}\subset K$ such that for any compact set $K'\subset K\setminus \{p_1,\cdots ,p_\ell \}$,

$$\begin{aligned} \lim _{i\rightarrow \infty } t_i^2||m_i||_{L^\infty (K')} = 0. \end{aligned}$$

In particular, for any $x\in K\setminus \{p_1,\cdots ,p_\ell \}$,

$$\begin{aligned} \lim _{i\rightarrow \infty }||F_{A_i}||_{L^\infty (E_x)} = 0. \end{aligned}$$

### Proof

Let $x_i$ be a sequence of points in *K* for which $t_i^2 m_i(x_i)$ does not approach zero. We will show a finite amount of energy must bubble off along this sequence. Thus, by the total energy bound (Lemma 3.3), there can only be a finite number of points in *K* where bubbling occurs.

The proof closely follows the arguments of [14, 17, 19, 21] and is divided into two cases. The first case occurs when $t_i^2m_i(x_i)$ is unbounded, and the second when $t_i^2m_i(x_i)$ stays bounded above, yet is also bounded away from zero. Unless mentioned otherwise, all bundle norms in this section are with respect to $H_0$, so we suppress $H_0$ from our notation for simplicity.

**Case 1** $t_i^2 m_i(x_i)$ is unbounded.

Given our sequence of points $x_i$, there exists corresponding points $a_i$ in $E_{x_i}$ where the supremum is obtained, and without loss of generality we can assume $(x_i,a_i)\rightarrow (x_0,a_0)$. Let $D_{r}(x_i)$ denote a disc of radius *r* in the metric $g_{{\mathbb {P}}^1}$ (corresponding to $\omega _{{\mathbb {P}}^1}$) in the base. We will show there exists a universal constant $\epsilon _0>0$, so that the inequality

4.1 $$\begin{aligned} \liminf _{i\rightarrow \infty }\int _{\pi ^{-1}(D_r(x_i))}|F_{\Xi _i}|^2_{g_i}dV_{g_i}>\epsilon _0 \end{aligned}$$

holds for any small $r>0.$ Since the total energy is finite, a standard covering argument then shows that there can only be finitely many bubbles of this type.

Suppose (4.1) does not hold. Then there exists an $r_0$ so that for sufficiently large *i*,

$$\begin{aligned} \int _{\pi ^{-1}(D_{r_0}(x_i))}|F_{\Xi _i}|^2_{g_i}dV_{g_i}\le \epsilon _0. \end{aligned}$$

It follows from (2.6) that there is a universal constant $c>0$, so that the $g_i$-geodesic ball $B_i :=B_{cr_0 {t_i}}(x_i,a_i)$ is contained in $\pi ^{-1}(D_{r_0}(x_i))$. In particular we have the following bound

$$\begin{aligned} \int _{B_i}|F_{\Xi _i}|^2_{g_i}dV_{g_i}\le \epsilon _0. \end{aligned}$$

We now rescale our coordinates and metrics. Consider the coordinate change

$$\begin{aligned} \uplambda _i( x,y) = \Big ({t_i}~( x+x_i),y+a_i\Big ), \end{aligned}$$

and let ${\tilde{\omega }}_i= t_i^{-2}\uplambda _i^*\omega _i$. To compare this to the scaling $L_{t_i}$, defined in (2.7) in Sect. 2, note that pulling the metric back by $L_{t_i}$ dilates the shrinking fibers, while ${\tilde{\omega }}_i$ is a combination of shrinking the base coordinates and then dilating the metric. Thus both scalings have the same effect, although with different coordinates.

By (2.6), ${\tilde{\omega }}_i$ is uniformly equivalent to the Euclidean metric in the scaled coordinates, which we denote by ${\tilde{g}}_0$. Moreover the ball $B_i$ pulls back to a ${\tilde{g}}_i$-geodesic ball ${\tilde{B}}_i$, which contains a Euclidean ball ${\tilde{B}}.$ The ball ${\tilde{B}}$ can be chosen to have uniform size independent of *i*.

Now, if ${\tilde{\Xi }}_i$ is the pull-back connection to these new coordinates, then the HYM equation is again satisfied

4.2 $$\begin{aligned} F_{{\tilde{\Xi }}_i}\wedge {\tilde{\omega }}_i = 0\qquad \mathrm{and}\qquad (F_{{\tilde{\Xi }}})^{0,2}=0. \end{aligned}$$

Change of variables, and the scale invariance of the Yang–Mills energy in dimension four, implies

$$\begin{aligned} ||F_{\tilde{\Xi }_i}||^2_{L^2(\tilde{B},\tilde{g}_0)}\le ||F_{\tilde{\Xi }_i}||^2_{L^2(\tilde{B}_i,\tilde{g}_i)}= ||F_{ \Xi _i}||^2_{L^2(B_i, g_i)} \le \epsilon _0. \end{aligned}$$

Since $\tilde{g}_i$ is uniformly equivalent to $\tilde{g}_0$ on ${\tilde{B}}$ for large *i*, and $\tilde{\Xi }_i$ satisfies (4.2), we can apply the standard $\epsilon$-regularity argument for Yang–Mills connections on a fixed ball $\tilde{B}$ [35, Theorem 4.8]. Thus, for $\epsilon _0$ small enough (depending only on the real dimension 4 of X), the above $L^2$ control implies

$$\begin{aligned} |F_{\tilde{\Xi }_i}|^2_{\tilde{g}_{i}}(0)\le C, \end{aligned}$$

for some constant *C* independent of *i*. Equivalence of metrics implies control of $F_{\tilde{\Xi }_i}$ with respect to ${\tilde{g}}_0$, which in components gives the following bound

$$\begin{aligned} |F_{\tilde{B}_i}|_{\tilde{g}_0}(0) + |F_{\tilde{A}_i}|_{\tilde{g}_0}(0) + |\tilde{\kappa }_i|^2_{\tilde{g}_0}(0)\le C. \end{aligned}$$

Scaling back, we see

$$\begin{aligned} t_i^2|F_{B_i}|_{g_X}(0) + |F_{A_i}|_{g_X}(0) +t_i^2|\kappa _i|^2_{g_X}(0)\le C. \end{aligned}$$

Hence we achieve control of $t_i^2 m_i(0)$, which we have assumed diverges, a contradiction. Let $W_1$ denote the set of points in *K* at which bubbles of this type appear.

**Case 2** $t_i^2m_i(x_i)$ is bounded above and away from zero.

In this case an instanton on ${{\mathbb {C}}}\times E$ bubbles off. We follow the outline of [19, 21], and use an energy quantization result of Wehrheim.

Suppose $x_i\rightarrow x_0 \in K\backslash W_1$, and let $D_{2\rho }(x_i)$ denote a disc of radius $2\rho$ in the metric $g_{{\mathbb {P}}^1}$ in the base. Suppose there exists constants $\delta$ and $\Lambda$ so that

$$\begin{aligned} \delta<t_i^2 m_i(x_i)\le \sup _{D_{2\rho }(x_i)}t_i^2 m_i<\Lambda . \end{aligned}$$

The rightmost inequality holds since for large enough *i* we can assume $D_{2\rho }(x_i)\subset K\backslash W_1$. By making $\rho$ smaller if necessary, we can furthermore assume that $\pi ^{-1}(D_{2\rho }(x_i))$ is topologically a product between a ball in ${\mathbb {C}}$ and an elliptic curve *E*, although the complex structure may vary.

We preform the same scaling as in Case 1, and define ${\tilde{\omega }}_i= t_i^{-2}\uplambda _i^*\omega _i$. Again this involves rescaling the metric and applying a dilation. The disk $D_{2\rho }(x_i)$ pulls back to $\tilde{D}_{{2\rho }/{{t_i}}}(0)$, the geodesic disk with respect to the Euclidean metric $\tilde{g}_0$ in the scaled coordinates. Starting from Proposition 2.4, the arguments used in the proof of [36, Theorem 1.1] (cf. pages 2936-2937) give that ${\tilde{\omega }}_i$ converge sub-sequentially and smoothly to a limiting flat product metric $\omega _\infty$ on ${\mathbb {C}}\times E$.

Our sequence of scaled connections $\tilde{\Xi }_i$ is defined on $\pi ^{-1}(\tilde{D}_{{2\rho }/{ {t_i}}}(0))$, and for any point $p\in \tilde{D}_{{2\rho }/{ {t_i}}}(0)$, we have

$$\begin{aligned} |F_{\tilde{B}_i}|_{\tilde{g}_0}(p) + |F_{\tilde{A}_i}|_{\tilde{g}_0}(p)+ |\tilde{\kappa }_i|^2_{ \tilde{g}_0}(p)&=t_i^2 |F_{B_i}|_{ g_0}(p)+|F_{A_i}|_{g_0}(p)+t_i^2|\kappa _i|^2_{ g_0}(p)\\&<\sup _{D_{2\rho }(x_i)}t_i ^2m_i < \Lambda . \end{aligned}$$

This implies $| F_{\tilde{\Xi }_i}|_{\tilde{g}_i}$ is uniformly bounded. By strong Uhlenbeck compactness [37, Corollary 1.4 and Theorem 1.5], this bound implies there exists a subsequence of connections which converges smoothly, modulo unitary gauge transformations, to a limiting connection $\tilde{\Xi }_\infty$ on the trivial *SU*(*n*) bundle over ${\mathbb {C}}\times E$. The connection ${\tilde{\Xi }}_\infty$ will be ASD with respect to the limiting product metric $\omega _\infty$. Furthermore, by assumption, we have

$$\begin{aligned} |F_{\tilde{B}_i}|_{\tilde{g}_0}(0)+|F_{\tilde{A}_i}|_{\tilde{g}_0}(0)+ |\tilde{\kappa }_i|^2_{ \tilde{g}_0}(0)>\delta , \end{aligned}$$

and it follows that the limiting connection is not flat. An energy quantization result of Wehrheim [38, Remark 1.2] implies there exists a universal constant $\epsilon _0>0$ so that

$$\begin{aligned} {\mathcal {Y}}{\mathcal {M}}(\tilde{\Xi }_\infty ,\tilde{g}_0)\ge 2\epsilon _0. \end{aligned}$$

This implies that there exists an $R>0$, so that for *i* sufficiently large,

$$\begin{aligned} \epsilon _0<\int _{\tilde{D}_{\frac{R}{2}}(0)\times E}|F_{\tilde{\Xi }_i}|^2_{\tilde{g}_i}dV_{\tilde{g}_i}=\int _{\pi ^{-1}(D_{R{t_i}}(x_i))}|F_{\Xi _i}|^2_{g_i}dV_i. \end{aligned}$$

Thus there can be only finitely many bubbles of this type, and denote the set of all such bubbles by $W_2$. This concludes the proof of Proposition 4.1. $\square$

We conclude this section by noting that Proposition 4.1, in conjunction with our convergence argument in Sect. 6, implies that on a generic fiber, the connections $A_i$ will converge to a limiting flat connection $A_0$. The following section is devoted to identifying this limiting flat connection explicitly.

## Gauge Fixing over an Elliptic Curve

In this section we work on a fixed fiber of $\pi$, denoted *E* for simplicity, satisfying $V|_E\cong \bigoplus _{j=1}^n {\mathcal {O}}_E(q_j-0)$ with each $q_j$ distinct and $q_1+\cdots +q_n=0$. By the results of Sect. 3, if *V* defines a spectral cover $D_V$ which is reduced and irreducible, this happens generically.

Equip *E* with the fixed Kähler form $$\omega _0=dy^1\wedge dy^2$$ and let $$g_0$$ denote the corresponding metric. Recall that *E* carries the complex coordinate $$z=\tau y^1+y^2$$. Denote the restriction $$ V|_E$$ by $$ V_0$$. Since $$V_0$$ is of the form $$\bigoplus _{j=1}^n {\mathcal {O}}_E(q_j-0)$$, it can be naturally identified with $$E\times {\mathbb {C}}^n$$, equipped with the complex structure

$$\begin{aligned} {\bar{\partial }}_{A_0}:={\bar{\partial }}-\frac{\pi \,Q}{\mathrm{Im}(\tau )}\,d{\bar{z}}, \end{aligned}$$

where *Q* is a diagonal matrix with entries $\tilde{q}_i$ (recall $\tilde{q}_i$ are the lifts of the points $q_i$ to ${\mathbb {C}}$). Let $H_0$ be the trivial metric on ${\mathbb {C}}^n$, and let $A_0$ be the Chern connection associated to ${\bar{\partial }}_{A_0}$ and $H_0$. Using (3.3), in addition to $dz|_E =\tau dy^1+dy^2$, one can explicitly check that that $A_0=\Xi _0|_E$, where $\Xi _0$ is given by (3.6).

Now, given our sequence of connections $$\Xi _i$$ on *V*, we have the sequence of restricted connections $$A_i:=\Xi _i|_E$$ on $$ V_0$$. Again, because our sequence of connections arises from the Donaldson–Uhlenbeck–Yau Theorem, we know that $$A_i$$ lies in the complexified gauge orbit of $$A_0$$, and thus $$A_i$$ and $$A_0$$ define isomorphic complex structures. As a result, after transforming $$A_0$$ by a unitary gauge transformation if necessary, we can write $$A_i=e^{s_i}( A_0)$$ for a trace free Hermitian endomorphism $$s_i$$. The main result of this section is:

### Theorem 5.1

Let $$e^s(A_0)$$ be a connection on $$ V_0$$ given by the action of a trace free Hermitian endomorphism *s*. There exists constants $$\epsilon _0>0$$, and $$C_0>0$$, depending only on $$g_0$$, $$A_0,$$ and $$H_0$$, so that the following holds. If the curvature of $$e^s(A_0)$$ satisfies

$$\begin{aligned} \qquad ||F_{e^s(A_0)}||^2_{C^0(g_0, H_0)}\le \epsilon _0, \end{aligned}$$

then there exists another trace free Hermitian endomorphism $s'$ which satisfied both

$$\begin{aligned} e^s( A_0)=e^{s'} ( A_0)\qquad \mathrm{and}\qquad ||s'||_{C^0(g_0, H_0) }\le C_0. \end{aligned}$$

Because $$ V_0$$ is poly-stable, and not stable, the above theorem is the best $$C^0$$ control that one can expect. For example, since $$A_0$$ is flat, if $$e^s$$ is a diagonal matrix of constants $$c_1,\ldots , c_n$$, then $$e^s ( A_0)$$ will still be flat. However, one eigenvalue $$c_i$$ can be arbitrarily large while still preserving the condition det$$(e^s)=1$$ (recall that $$e^s\in SL( V_0)$$). Thus, one can never expect $$C^0$$ control for *s*. The main idea of the above theorem is that, by a suitable choice of normalization, one can construct a related complex gauge transformation that yields the same connection, yet with the desired $$C^0$$ control.

We first demonstrate several preliminary results. For the remainder of the section, unless specified, all norms are taken with respect to the metrics $g_0$ and $H_0$, and we remove this from our notation for simplicity.

### Proposition 5.2

Let *s* be a trace free Hermitian endomorphism such that the diagonal entries of *s* have zero average when integrated over *E*. Then there exists a constant $C_p$, independent of *s*, so that

5.1 $$\begin{aligned} ||s||_{L^2(E)}\le C_p||{\bar{\partial }}_{A_0}s||_{L^2(E)}. \end{aligned}$$

### Proof

Note that diagonal entries of *s* can always be defined as the entries that preserve each subbundle ${\mathcal {O}}_E(q_i-0)\subset V_0$. Now, assume that the inequality does not hold. Then there exists a sequence of endomorphisms $s_k$ satisfying the assumptions of the proposition, along with the inequality

$$\begin{aligned} \int _E|{\bar{\partial }}_{A_0} s_k|^2\le \frac{1}{k}\int _E |s_k|^2. \end{aligned}$$

Let $$\tilde{s}_k:=s_k/||s_k||_{L^2(E)}$$. Then

$$\begin{aligned} \int _E|{\bar{\partial }}_{A_0} \tilde{s}_k|^2\le \frac{1}{k}. \end{aligned}$$

Because *s* is Hermitian with respect to $H_0$, we have $|{\bar{\partial }}_{A_0} \tilde{s}_k|=|\partial _{A_0} \tilde{s}_k|$. This allows us to conclude that the sequence $\tilde{s}_k$ converges weakly in $L^2_1$ (and strongly in $L^2$) to an endomorphism $s_\infty$ satisfying

$$\begin{aligned} ||s_\infty ||_{L^2(E)}=1\qquad \mathrm{and}\qquad ||{\bar{\partial }}_{A_0} s_\infty ||_{L^2(E)}=0. \end{aligned}$$

Now, because *Q* is diagonal, if $$s_\infty $$ has entries $$(a_{ij})$$, then the diagonal entries of $${\bar{\partial }}_{A_0} s_\infty $$ are of the form $${\bar{\partial }} \,a_{ii}$$. Since $$||{\bar{\partial }}_{A_0} s_\infty ||_{L^2}=0$$, we see the diagonal entries of $$s_\infty $$ are constant. Furthermore, by assumption the diagonal entries of $$\tilde{s}_k$$ have zero average, and by strong convergence in $$L^2$$ we conclude the diagonal entries of $$s_\infty $$ must also have zero average. Thus these entries vanish entirely. Now, because the points $$q_i$$ are distinct, the automorphism group of $$ V_0$$ is precisely *n* dimensional [16, Lemma 1.13]. Thus if the diagonal entries of $$s_\infty $$ vanish, $$s_\infty $$ must vanish entirely. In other words, if $$s_\infty $$ had any non-vanishing off diagonal entries, they would define a holomorphic map between line bundles $$ {\mathcal {O}}_E(q_i-0)$$ and $$ {\mathcal {O}}_E(q_j-0)$$ for distinct points $$q_i$$ and $$q_j$$, which is impossible. So $$s_\infty =0$$ yet $$||s_\infty ||_{L^2(E)}=1$$, a contradiction. $$\square $$

Next consider the following function spaces, equipped with the $L^2$ norm.

### Definition 5.3

Let $$\mathrm{Herm}_0( V_0)$$ be the space of trace free Hermitian endomorphisms of $$ V_0$$, and furthermore let $$\text {Herm}^{\perp _{0}}( V_0)$$ denote the subspace consisting of those endomorphisms whose diagonal entries have zero average on *E*.

Consider $\Upsilon (\cdot )\in \text {End}({{\mathfrak {g}}l}( V_0))$, defined by

$$\begin{aligned} \Upsilon (s)=\frac{e^{\mathrm{ad}_s}-1}{\mathrm{ad}_s}. \end{aligned}$$

From the definition of the complexified gauge action (3.1), we have

5.2 $$\begin{aligned} \partial _{e^{s}( A_0)}=\partial _{A_0}+e^{-s}(\partial _{A_0} e^{s})\qquad \mathrm{and}\qquad {\bar{\partial }}_{e^{s} ( A_0)}={\bar{\partial }}_{A_0}-({\bar{\partial }}_{A_0} e^{s})e^{-s}. \end{aligned}$$

This allows one to compute

5.3 $$\begin{aligned} e^{s} (A_0)=A_0+\Upsilon ({-s})\partial _{A_0} s-\Upsilon ({s}){\bar{\partial }}_{A_0} s \end{aligned}$$

(for instance, see [39, Appendix A]).

Define the map ${{\mathcal {N}}(s)}:=e^{s} (A_0)$, which maps $\mathrm{Herm}_0( V_0)$ into the affine space of connections centered at $A_0$, equipped with the $L^2$ norm. Let ${{\mathcal {A}}}$ denote the image of the map ${\mathcal {N}}$. Using (5.3), we see the derivative of ${\mathcal {N}}$ at 0 is given by

$$\begin{aligned} L(s):={{\mathcal {N}}}'(0)(s)=\partial _{A_0} s-{\bar{\partial }}_{A_0} s. \end{aligned}$$

The tangent space to $\mathrm{Herm}_0( V_0)$ is again $\mathrm{Herm}_0( V_0)$. Note that for any $s\in \mathrm{Herm}_0( V_0)$, if ${\hat{s}}$ is a diagonal matrix of constants given by the averaging the diagonals of *s* over *E*, then $L(s)=L(s-{\hat{s}})$, and so both $\mathrm{Herm}_0( V_0)$ and $\mathrm{Herm}^\perp _0( V_0)$ have the same image under *L*. Proposition 5.2 shows that *L* has trivial Kernel on $\mathrm{Herm}^\perp _0( V_0)$. Thus, not only can we conclude that the restriction of *L* to $\mathrm{Herm}^\perp _0( V_0)$ is invertible, but (5.1) shows in addition that *L* has bounded inverse. The contraction mapping principle implies there exists a small neighborhood ${{\mathcal {U}}}\subset {{\mathcal {A}}}$ of the connection $A_0$, and a set ${\mathcal {V}}\subset \mathrm{Herm}^\perp _0( V_0)$ in the tangent space to $\mathrm{Herm}_0( V_0)$ at 0, so that ${{\mathcal {V}}}\longrightarrow {{\mathcal {U}}}$ is a diffeomorphism onto its image.

Summing up, we have proved the following:

### Lemma 5.4

There exist constants $\delta _0$ and $\Lambda _0$, which depend only on $A_0$, $H_0$ and $g_0$, so that the following holds. If $A\in \mathcal {A}$ satisfies $||A - A_{0}||_{L^2(E)}< \delta _0$, there exists $s\in \mathrm{Herm}_0^\perp$, such that

$$\begin{aligned} A = e^{s} (A_{0}), \end{aligned}$$

and

$$\begin{aligned} ||s||_{L^2(E) }\le \Lambda _0||e^{s} (A_0)-A_0||_{L^2(E) }. \end{aligned}$$

We now turn to one final lemma. Consider the same constant $\delta _0>0$ from above, and let $C_0>0$ be a fixed constant, to be determined in the proof of Theorem 5.1.

### Lemma 5.5

Let *s* be a trace free Hermitian endomorphism, and *A* a flat connection on $$ V_0$$. Given constants $$\delta _0>0$$ and $$C_0>0$$, there exists a constant $$\epsilon _0$$, depending only on $$H_0$$, $$g_0$$, $$\delta _0$$, and $$C_0$$, so that if $$||s||_{C^0 }\le C_0$$ and $$||F_{e^{s} (A)}||_{C^0(E)}<\epsilon _0$$, then

$$\begin{aligned} ||e^{s} (A)-A||_{L^2(E)}<\frac{\delta _0}{2}. \end{aligned}$$

### Proof

To begin, we see how the curvature of *A* is related to the curvature of $e^{s} (A)$. Using (5.2) one can compute

$$\begin{aligned} e^{-s}F_{e^{s} (A)} e^{s}-F_{A}={\bar{\partial }}_A(e^{-2s}\partial _A e^{2s})=e^{-2s}\left( {\bar{\partial }}_A\partial _A e^{2s}-{\bar{\partial }}_Ae^{2s} e^{-2s}\partial _A e^{2s}\right) , \end{aligned}$$

which implies

5.4 $$\begin{aligned} -\Delta _{g_0}\mathrm{Tr}(e^{2s})+|e^{-s}\partial _A e^{2s}|^2_{g_0}=\mathrm{Tr}(e^{2s}i\Lambda _{\omega _0} F_{e^{s} (A)}). \end{aligned}$$

Integrating over *E* yields

$$\begin{aligned} \int _E |e^{-s}\partial _A e^{2s}|^2<e^{C_0}\epsilon _0. \end{aligned}$$

The $C_0$ bound for *s*, along with det$(e^s)=1$, demonstrates the eigenvalues of $e^s$ are bounded above and below. Thus the left hand side above controls the $L^2$ norm of the difference $e^{s}(A)-A$, and so

$$\begin{aligned} ||e^{s}(A)-A||_{L^2(E)}<C\epsilon _0. \end{aligned}$$

Here *C* only depends on $C_0$, $g_0$, and $H_0$. Choose $\epsilon _0$ small so $C\epsilon _0<\delta _0/2$. $\square$

As we turn to the proof of Theorem 5.1, recall that the constants $\delta _0$ and $\Lambda _0$ depend only on $A_0$, $H_0$ and $g_0$. Thus, by the above lemma, if we can show $C_0$ depends only on these quantities, $\epsilon _0$ will depend only on these quantities.

### Proof of Theorem 5.1

Our first task is to specify $C_0$. Fix an endomorphism $s\in \mathrm{Herm}_0( V_0)$ satisfying

$$\begin{aligned} \qquad ||F_{e^s(A_0)}||^2_{C^0}\le \epsilon _0. \end{aligned}$$

Using this curvature bound, along with the inequality

$$\begin{aligned} -\Delta _{g_0}|s|^2\le |s| |F_{e^{s} (A_0)}| \end{aligned}$$

(see for instance Proposition A.6 in [39]), we can apply Moser iteration to conclude

5.5 $$\begin{aligned} \max \{|s|^2,1\}\le C_1||s||_{L^2 }(1+\epsilon _0). \end{aligned}$$

Here $C_1$ only depends on $g_0$ and $H_0$. We now set $C_0:= 2C_1\Lambda _0$. This shows $C_0$, and subsequently $\epsilon _0$, depends only on the initial setup.

The main idea of the proof is as follows. We construct a path of Hermitian endomorphisms, so that the curvature of the induced connections along this path is bounded by $\epsilon _0$. We show the endpoint of our path satisfies the conclusion of the theorem, and then apply a method of continuity argument to conclude our desired result for *s*. Naively, one may first try to connect $e^{s}$ to $Id_{V_0}$ by the path $e^{ts}$ for $t\in [0,1]$. However, for arbitrary initial *s* it is not clear that curvature stays bounded by $\epsilon _0$ along this path, which is an important for the argument. Instead, we follow the Yang–Mills flow.

Following Donaldson [12, Sect. 1.1], we consider a path of complex gauge transformations *g*(*t*), satisfying

$$\begin{aligned} \dot{g}(t) g(t)^{-1}=-i\star F_{g(t) (A_0)}\qquad \qquad g(0)=e^{s}. \end{aligned}$$

On a Riemann surface, the above flow is referred to as the Kempf–Ness flow, and given a solution, the corresponding connections $A(t):=g(t) (A_0)$ solve the Yang–Mills heat flow:

$$\begin{aligned} \dot{A}(t)=-d^*_{A(t)}F_{A(t)}\qquad \qquad A(0)=e^{s} (A_0). \end{aligned}$$

By a Theorem of Rade [40, Theorem 2], there exists a limiting Yang–Mills connection $A_\infty$ for which

$$\begin{aligned} ||A(t)-A_\infty ||_{L^2_1}\le c\,t^{-\beta }. \end{aligned}$$

Since $$ V_0$$ is polystable, the limit connection $$A_\infty $$ is also flat, and in the unitary gauge orbit of $$A_0$$, and so there exists a unitary gauge transformation $$u_\infty $$ for which $$A_\infty =u_\infty ^* A_0$$.

Consider the trivial flow $u_\infty (t)=u_\infty$, which again satisfies the Kempf–Ness flow equation

$$\begin{aligned} \dot{u}_\infty (t)u_\infty (t)^{-1}=-i\star F_{u_\infty (t) ( A_0)}=-i\star F_{A_\infty }=0. \end{aligned}$$

Define $\eta (t)\in \mathrm{Herm}_0( V_0)$, and a path of unitary gauge transformations *u*(*t*), by the equation

5.6 $$\begin{aligned} u_\infty u(t)e^{\eta (t)}=g(t). \end{aligned}$$

Thus, $u(t)e^{\eta (t)}$ relates our two solutions of the Kempf–Ness flow. By Proposition 4.13 in [41], both *u*(*t*) and $\eta (t)$ are bounded in $L^2_2$. In fact, this $L^2_2$ bound is proven following the general argument of [42]. The authors demonstrate that if *M* is a complete, connected, simply connected Riemannian manifold of nonpositive sectional curvature, which admits a function $\Phi :M\rightarrow {\mathbb {R}}$ which is convex along geodesics, then given two negative gradient flow lines of $\Phi$, the geodesic distance between these two flow lines stays bounded. In our case, the role of *M* is taken by the space of Hermitian metrics, and the function $\Phi$ is Donaldson’s functional (see [12, 41, 42], for a precise definition of $\Phi$).

The bound on $\eta (t)$, along with convergence of $F_{A(t)}$ to zero in $L^2$, allows us to conclude by Lemma 5.5 that there exists a *T* sufficiently large, so that

$$\begin{aligned} ||(u_\infty u(T) e^{\eta (T)}u(T)^{-1}u_\infty ^{-1})\left( (u_\infty u(T))^*A_0\right) -(u_\infty u(T))^*A_0 ||_{L^2}\le \frac{\delta _0}{2}. \end{aligned}$$

For simplicity we denote the fixed unitary gauge transformation $u_\infty u(T)$ by *u*, and define the path of Hermitian endomorphism $e^{\kappa (t)}$ by $u e^{\eta (t)}u^{-1}$. Then the above estimate can be written

5.7 $$\begin{aligned} ||e^{\kappa (T)} (u^*A_0)-u^*A_0 ||_{L^2}\le \frac{\delta _0}{2}. \end{aligned}$$

It is along the path $e^{\kappa (t)}$ that we can now apply our method of continuity argument.

Let $\tilde{A}(t)$ be the path of connections given by $e^{\kappa (t)} (u^*A_0)$. Since $\tilde{A}(t)=(u_\infty u(t)u^{-1})^*A(t)$, where *A*(*t*) solves the Yang–Mills flow and *u*(*t*) is given by (5.6), we conclude that $||F_{\tilde{A}(t)}||_{C^0(E)}\le \epsilon _0$ for all $t\in [0,T]$. This follows because the curvature is decreasing along the Yang–Mills flow, and the action of a unitary gauge transformation will not affect this norm. Also, the path $\tilde{A}(t)$ is smooth for $t\in [0,T]$.

To set up the method of continuity, consider the set $I\subseteq [0,T]$ consisting of times *t* for which there exists a trace free Hermitian endomorphism $\kappa '(t)$ which satisfies both

5.8 $$\begin{aligned} e^{\kappa '(t)} (u^*A_0)=e^{\kappa (t)} (u^*A_0) \end{aligned}$$

and

5.9 $$\begin{aligned} ||\kappa '(t)||_{C^0(g_0,H_0)}\le C_0. \end{aligned}$$

We prove $I=[0,T]$. First, we demonstrate $T\in I$ to conclude *I* is non-empty. By the estimate (5.7), we can apply Lemma 5.4 to $\tilde{A}(T)$, and conclude there exists a trace free Hermitian endomorphism $\kappa '(T)$ satisfying both (5.8) and an $L^2$ bound. By our Moser iteration bound this $L^2$ control can be improved to $C_0,$ and so $\kappa '(T)$ satisfies (5.9) as well. Thus $T\in I$. We now need that *I* is both open and closed with respect to the topology induced from the $C^2$ topology on Herm$( V_0)$. For the rest of the proof we use the notation $A:= u^*A_0$.

Our next step is to show *I* is open. Let $t_0\in I$, and consider the corresponding endomorphism $e^{\kappa _0}$. Construct a small neighborhood of $e^{\kappa _0}$ with radius $\rho >0$, where $\rho$ is chosen so

$$\begin{aligned} ||e^{\kappa }-e^{\kappa _0}||_{C^2(E) }<\rho \end{aligned}$$

implies

$$\begin{aligned} ||e^{\kappa } ( A) - e^{\kappa _0} ( A)||_{L^{2}(E) } < \delta _0/2. \end{aligned}$$

Now, because $e^{\kappa _0}\in I$, there exists an endomorphism $e^{\kappa '_0}$ satisfying both $e^{\kappa _0} ( A)= e^{\kappa _0'} (A)$ and $||\kappa _0'||_{C^0 } \le C_0.$ Given our choice of $\epsilon _0$, by Lemma 5.5 we have

$$\begin{aligned} ||e^{\kappa _0}(A)- A||_{L^2 } =||e^{\kappa '_0}(A)- A||_{L^2 } < \delta _0/2. \end{aligned}$$

By the triangle inequality $||e^{\kappa } ( A_0) - A_{0}||_{L^2 } < \delta _0$, and thus Lemma 5.4 implies there exists an endomorphism $\kappa '\in \mathrm{Herm}_0^\perp$ such that $e^{\kappa } ( A)=e^{\kappa '} ( A)$ and

$$\begin{aligned} ||\kappa '||_{L^2 }\le \Lambda _0||e^{\kappa '} ( A)-A||_{L^2 }<\Lambda _0 \end{aligned}$$

(we assumed $\delta _0<1$). By our Moser iteration bound (5.5), we conclude

$$\begin{aligned} ||\kappa '||_{C^0 }<2\Lambda _0C_1 =C_0, \end{aligned}$$

which completes the proof of openness.

Finally we prove *I* is closed. Let $t_i$ be a sequence of times in *I* converging to *t*, and let $\kappa _i$ be the corresponding sequence of endomorphisms converging to $\kappa$ in the $C^2$ topology. For each *i*, there exists $\kappa _i'$ which are uniformly bounded in $C^0$ and satisfy $e^{\kappa _i} ( A)= e^{\kappa _i'} (A)$. The complexified gauge action gives

$$\begin{aligned} e^{ \kappa _i}\circ {\bar{\partial }}_{ A}\circ e^{- \kappa _i}=e^{ \kappa _i'}\circ {\bar{\partial }}_{ A}\circ e^{- \kappa _i'}, \end{aligned}$$

from which we conclude ${\bar{\partial }}_{A}(e^{- \kappa _i}e^{ \kappa _i'})=0$. Since $A=u^*A_0$, this implies ${\bar{\partial }}_{A_0}(u^{-1}e^{- \kappa _i}e^{ \kappa _i'}u)=0$. Since $A_0$ has only diagonal entries, we see the diagonal entries of $u^{-1}e^{- \kappa _i}e^{ \kappa _i'}u$ must be constant. As before it then follows that the off diagonal terms must vanish, otherwise one would have a holomorphic map between line bundles ${\mathcal {O}}_E(q_i-0)$ and ${\mathcal {O}}_E(q_j-0)$ for distinct points $q_i$ and $q_j$. Taking the complex conjugate and using the fact that $\kappa _i$ and $\kappa _i'$ are Hermitian, we see that $h_i:=u^{-1}e^{ \kappa _i'}e^{- \kappa _i}u$ will be a diagonal matrix of constants.

Now, $$C^2$$ convergence of $$ \kappa _i$$ together with the $$C^0$$ control of $$ \kappa _i'$$ gives that the matrices $$ h_i$$ are uniformly bounded above and below. Since each $$ h_i$$ is a diagonal matrix of constants, after passing to a subsequence the $$ h_i$$ converge in $$C^0$$ to a limit *h*. This allows us to define an endomorphism $$e^{ \kappa '}:= uh u^{-1}e^{ \kappa }$$, and by convergence of $$ h_i$$ and $$ \kappa _i$$ we have

$$\begin{aligned} e^{ \kappa _i'}= uh_iu^{-1} e^{ \kappa _i}\rightarrow e^{ \kappa '} \end{aligned}$$

in $C^0$. Since $e^{\kappa _i'}$ is Hermitian, so is $e^{ \kappa '}$, and thus $\kappa '$ is Hermitian and satisfies (5.9). Furthermore, since for each *i* we have ${\bar{\partial }}_A(u h_iu^{-1})=0$, we in fact have $u h_iu^{-1} e^{ \kappa _i}\rightarrow e^{ \kappa '}$ in $C^1$, and as a result we conclude $e^{ \kappa '} ( A)= e^{ \kappa } (A)$. Thus (5.8) is also satisfied and $t\in I$.

Thus, *I* is open, closed, and nonempty, and as a result $e^{\kappa (0)}\in I$. In particular, there exists a $\kappa '(0)$ satisfying both $e^{\kappa (0)} (A)= e^{\kappa (0)'} (A)$ and $||\kappa (0)'||_{C^0 } \le C_0.$ Now, define $s'$ by

$$\begin{aligned} e^{s'}=u^{-1}e^{\kappa (0)'}u. \end{aligned}$$

We see $s'$ satisfies the desired $C^0$ bound. Furthermore,

$$\begin{aligned} e^{s'} (A_0)=u^{-1}{}^*e^{\kappa (0)'} (u^*A_0)= u^{-1}{}^*e^{\kappa (0)} (u^*A_0)= & {} e^{\eta (0)} ( A_0). \end{aligned}$$

Yet we started the flow (5.6) at $g(0)=e^{s}$, so $\eta (0)=s$. This completes the proof of the theorem. $\square$

## Convergence

In this section, we complete the proof of Theorem 1.1. As before, let $Z_\pi$ denote image of the singular fibers under $\pi$, and $W_1$ and $W_2$ the bubbling sets for our sequence of connections. We fix a point $x\in {\mathbb {P}}^1\backslash (Z_\pi \cup W_1\cup W_2)$, and denote the fiber over *x* by $E:=\pi ^{-1}(x)$. We use the notation $A_i:=\Xi _i|_{E}$ and $A_0:=\Xi _0|_{E}$. As above equip *E* with the fixed flat metric $\omega _0=dy^1\wedge dy^2$. Unless otherwise specified, in this section all norms are taken with respect to the metrics $g_0$ and $H_0$.

Recall our bubbling sequence at *x* is defined by

$$\begin{aligned} m_i(x):=||F_{B_i}||_{C^0(E,g_X)}+\frac{1}{t_i^2}||F_{A_i}||_{C^0(E,g_X)}+||\kappa _i||^2_{C^0(E, g_X)}. \end{aligned}$$

Since $x\notin W_1\cup W_2$, we have $t_i^2 m_i\rightarrow 0$ as $i\rightarrow \infty$, so $||F_{A_i}||_{C^0(E)}\rightarrow 0$. As mentioned at the end of Sect. 4 (and as we shall see below), this is enough to prove that $A_i$ converges, along a subsequence and modulo gauge transformations, to a limiting flat connection. Our main result is identifying this limit.

Assume $V|_E= {\mathcal {O}}_E(q_1-0)\oplus \cdots \oplus {\mathcal {O}}_E(q_n-0)$ for $q_1,\ldots ,q_n$ distinct. Writing $A_i=e^{s_i}(A_0)$ for a sequence of Hermitian endomorphism $s_i$, for *i* large enough we can apply Theorem 5.1 to conclude there exists gauge transformations $s_i'$, which are uniformly bounded in $C^0$, and satisfy $A_i=e^{s_i'}( A_0)$. Thus, as in the proof of Lemma 5.5,

6.1 $$\begin{aligned} ||A_{i}-A_0||_{L^2(E)}\le C ||e^{-s'_i}\partial _{A_0}e^{2s'_i}||_{L^2(E)}\le C||F_{A_i}||_{L^2(E)}\rightarrow 0. \end{aligned}$$

Furthermore, since $A_0$ is flat, we can integrate by parts and change the order of derivatives to conclude:

$$\begin{aligned} ||\nabla ^0(A_i-A_0)||_{L^2(E)}^2= & {} \int _{E_p}\mathrm{Tr}(\nabla ^0(e^{-2s_i} \nabla ^0 e^{2s_i})\left( \nabla ^0(e^{-2s_i}\nabla ^0 e^{2s_i})\right) ^*)\\= & {} \int _{E_p}\mathrm{Tr}({\bar{\nabla }}^0(e^{-2s_i} \nabla ^0 e^{2s_i})\left( {\bar{\nabla }}^0(e^{-2s_i}\nabla ^0 e^{2s_i})\right) ^*)\\= & {} ||F_{A_i}||^2_{L^2(E)}\rightarrow 0. \end{aligned}$$

Thus we have demonstrated

$$\begin{aligned} ||A_{i}-A_0||_{L^2_1(E)}\rightarrow 0, \end{aligned}$$

which is the stated convergence in Theorem 1.1.

Next we prove smooth convergence, allowing for the action of unitary gauge transformations. Specifically, since *x* is away from the bubbling set, there exists a small disk $D_\rho (x)$ that does not intersect $W_1\cup W_2$. We use the same coordinate transformations $\uplambda _i$ in the proof Proposition 4.1 sending *x* to the origin and scaling. Consider the rescaled metrics $\tilde{\omega }_i=t_i^{-2}\uplambda _i^*\omega _{t_i}$. The set $\pi ^{-1}(D_\rho (x))$ rescales to a set topologically equivalent to $\tilde{D}_{\frac{\rho }{{ t_i}}}(0)\times E$ (although the complex structure will not be a product).

Our sequence of connections $\Xi _i$ pulls back to $\tilde{\Xi }_i$, with fiber and base components

$$\begin{aligned} \tilde{A}^i_j=A^i_j\qquad \mathrm{and}\qquad \tilde{B}^i_j= { t_i} B^i_j. \end{aligned}$$

As before, the connections $$\tilde{\Xi }_i$$ are HYM with respect to $$\tilde{\omega }_{ i}$$, and each $$\tilde{\omega }_i$$ is uniformly equivalent to the Euclidian metric for large *i* by Proposition 2.4. Also, since $$D_\rho (x)$$ is away from $$W_1$$, the function $$ t_i^2m_i(x)$$ is uniformly bounded above on the disk. This implies the curvature $$|F_{\tilde{\Xi }_i}|$$ is uniformly bounded on $$\pi ^{-1}(\tilde{D}_{\frac{\rho }{ { t_i}}}(0))$$.

Applying strong Uhlenbeck compactness [37, Corollary 1.4 and Theorem 1.5] on the fixed compact set

$$\begin{aligned} \pi ^{-1}(\tilde{D}_{1}(0))\subset \pi ^{-1}(\tilde{D}_{\frac{\rho }{{ t_i}}}(0)), \end{aligned}$$

there exists a sequence of gauge transformations $u_i$ so that along a subsequence, $u_i^* \tilde{\Xi }_i$ converges smoothly to a limiting Yang–Mills connection $\tilde{\Xi }_\infty$. Restricting our attention to the fiber *E* over the origin yields a sequence of connections $u_i^*\tilde{A}_i$ which converges smoothly to a limiting flat connection $\tilde{A}_\infty$. Note that our fiber coordinates are not scaled, and the restriction of $\tilde{\omega }_{ t_i}$ to *E* is equivalent the standard metric $\omega _0=dy^1\wedge dy^2$. Thus, on *E*, we see $u_i^*A_i$ converges smoothly to a flat connection $A_\infty$. The connection $A_\infty$ may not equal $A_0$, but it will lie in the unitary gauge orbit.

We conclude by remarking that estimates of the form (6.1) are common in these types of degeneration problems, for example see Proposition 3.1 in [18] or Theorem 1 in [43]. In our estimate, the curvature term is not raised to a power, and this holds because the specific form of our complex structure $V|_E= \oplus _{j=1}^n{\mathcal {O}}_E(q_j-0)$ implies that the Yang–Mills energy functional is Morse-Bott at $A_0$ (see Definition 7.5 in [43]). Essentially, the argument in our proof of Proposition 5.2 gives that the kernel of the Hessian operator of the Yang–Mills energy functional can be identified with one forms valued in constant diagonal matrices, which also gives the tangent space to Yang–Mills connections at $A_0$. We direct the reader to [43] for further details.
